# Supplementary material for: Dual-phase nano-glass-hydrides overcome the strength-ductility trade-off and magnetocaloric bottlenecks of rare earth based amorphous alloys
Source: Nat Commun. 2024 May 16;15:4159. doi: 10.1038/s41467-024-48531-7 (PMC11099109; doi:10.1038/s41467-024-48531-7)
Supplement: Supplementary file 1 — Supplementary Information [file 41467_2024_48531_MOESM1_ESM.pdf]

Supplementary Information

Dual-phase nano-glass-hydrides overcome the strength-ductility trade-off and magnetocaloric bottlenecks of rare earth based amorphous alloys

Liliang Shao[1,2,3], Qiang Luo[1*], Mingjie Zhang[1], Lin Xue[4], Jingxian Cui[1], Qianzi Yang[1], Haibo Ke[2*], Yao Zhang[1], Baolong Shen[1*], Weihua Wang[2,3]

[1]*School of Materials Science and Engineering, Jiangsu Key Laboratory of Advanced Metallic Materials, Southeast University, Nanjing 211189, China*

[2]*Songshan Lake Materials Laboratory, Dongguan 523808, China*

[3]*Institute of Physics, Chinese Academy of Sciences, Beijing 100190, China*

[4]*College of Mechanics and Materials, Hohai University, Nanjing 211100, China*

*These authors jointly supervised this work: Qiang Luo (E-mail: q.luo@seu.edu.cn), Haibo Ke (E-mail: kehaibo@sslab.org.cn) and Baolong Shen (E-mail: blshen@seu.edu.cn).

This file includes:

Supplementary Figure 1. Structural characterizations of the amorphous powders after hydrogenation.

Supplementary Figure 2. Mechanical properties of the GdNiAl(H) and DyCoAlSi(H) micropillars.

Supplementary Figure 3. Mechanical property of the GdCoAlH micropillars.

Supplementary Figure 4. Magnetocaloric measurements of the GdCoAl(H) powders.

Supplementary Figure 5. Magnetocaloric measurements of the GdNiAl(H) powders.

Supplementary Figure 6. Magnetocaloric measurements of the DyCoAlSi(H) powders.

Supplementary Figure 7. Magnetic behavior of the DyCoAlSi(H) powders.

Supplementary Figure 8. Universality of hydrogenation to improve magnetic entropy change (ΔS_M).

Supplementary Figure 9. Nanoindentation test of the GdCoAl(H) powders.

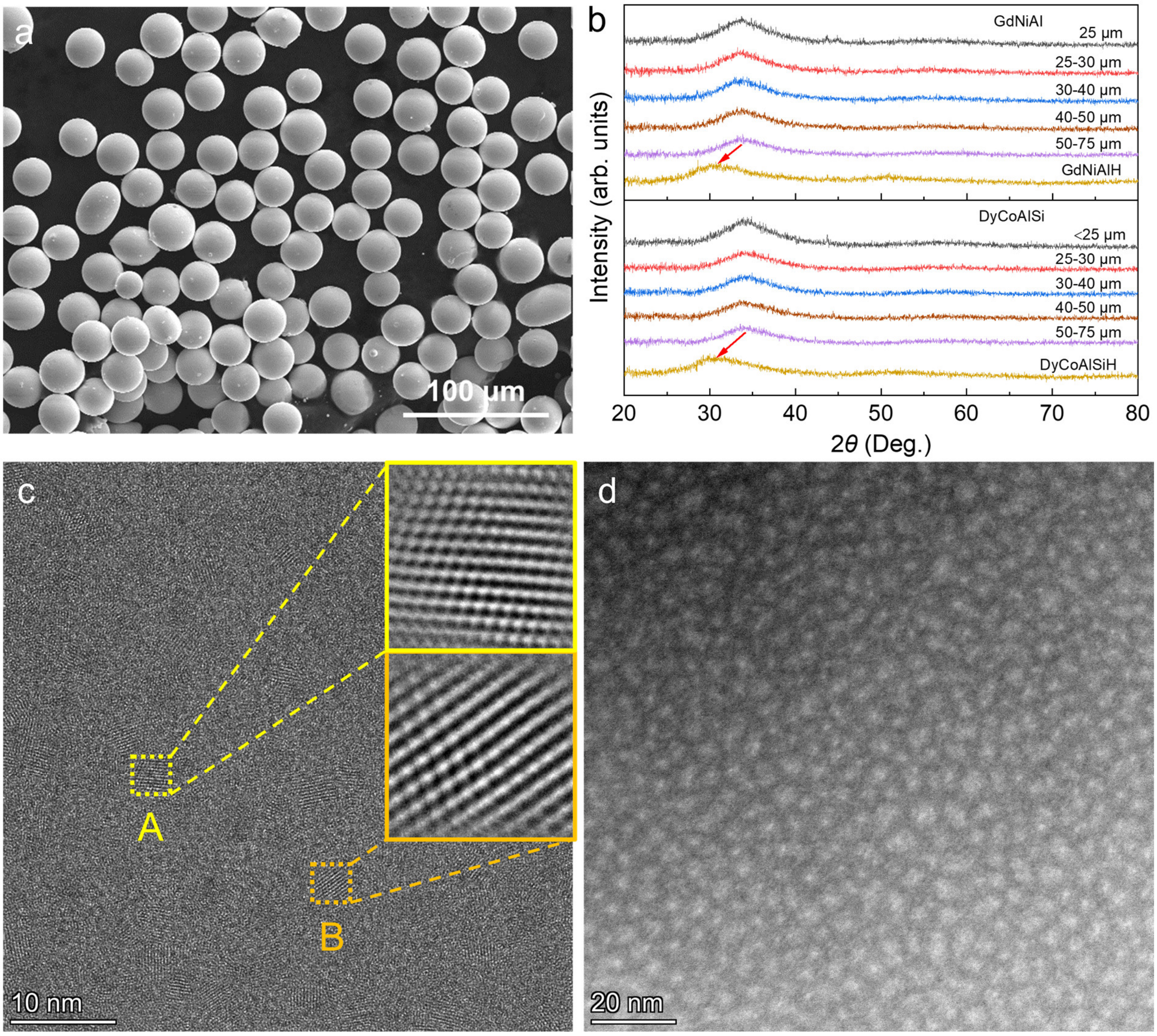

Supplementary Figure 1. Structural characterizations of the amorphous powders after hydrogenation. a Scanning electron microscope (SEM) image of the DyCoAlSi amorphous powders with the diameter of 25-30 μm. **b** X-ray diffraction (XRD) patterns of the GdNiAl and GdNiAlH as well as DyCoAlSi and DyCoAlSiH amorphous powders. **c** High-resolution transmission electron microscopy (HRTEM) and **d** high angle annular dark-field (HAADF) images of the DyCoAlSiH powders.

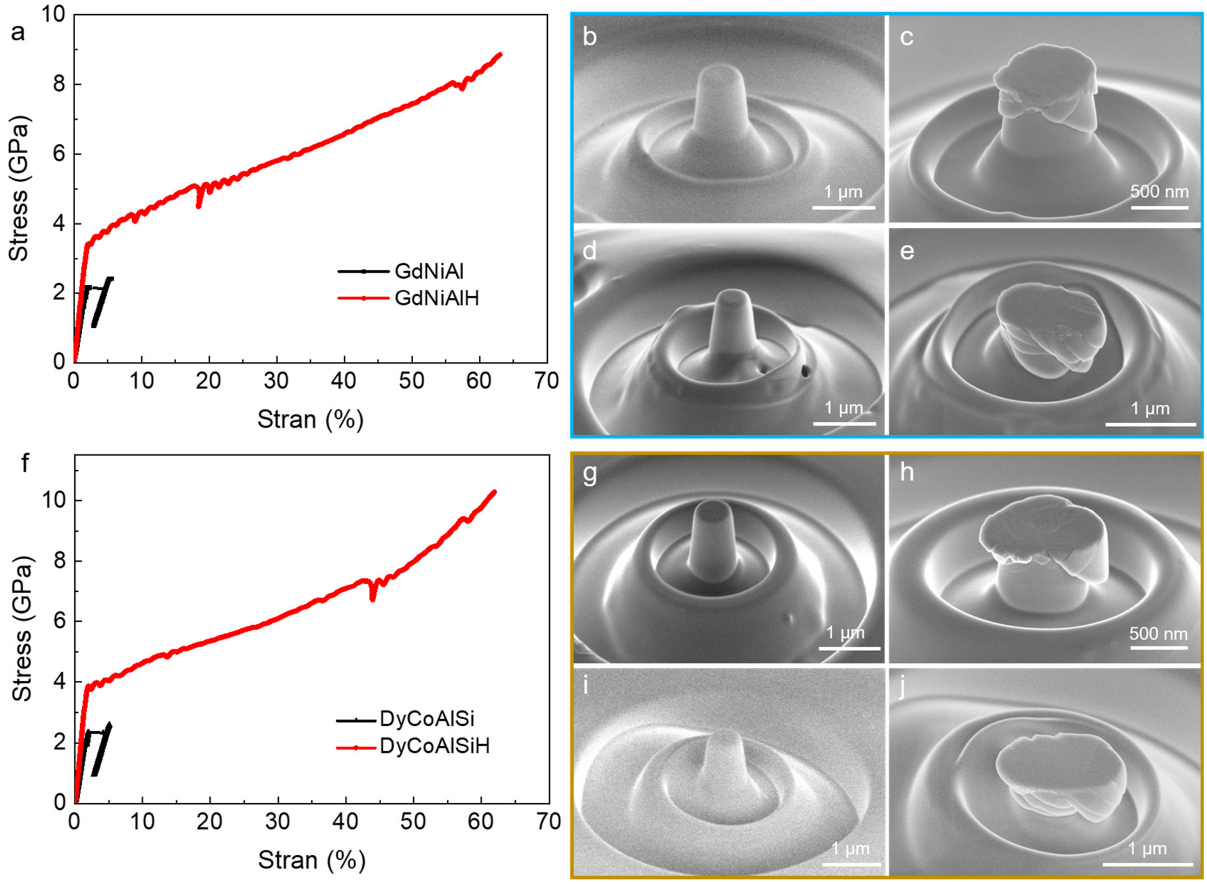

Supplementary Figure 2. Mechanical properties of the GdNiAl(H) and DyCoAlSi(H) micropillars. **a** Engineering stress-strain curves of the GdNiAl and GdNiAlH micropillars with a diameter of 500 nm and height of 1 μm. SEM images of the **b**, **c** GdNiAl and **d**, **e** GdNiAlH pillars before and after compression. **f** Engineering stress-strain curves of the DyCoAl and DyCoAlH micropillars with a diameter of 500 nm and height of 1 μm. SEM images of the **g**, **h** DyCoAl and **i**, **j** DyCoAlH pillars before and after compression.

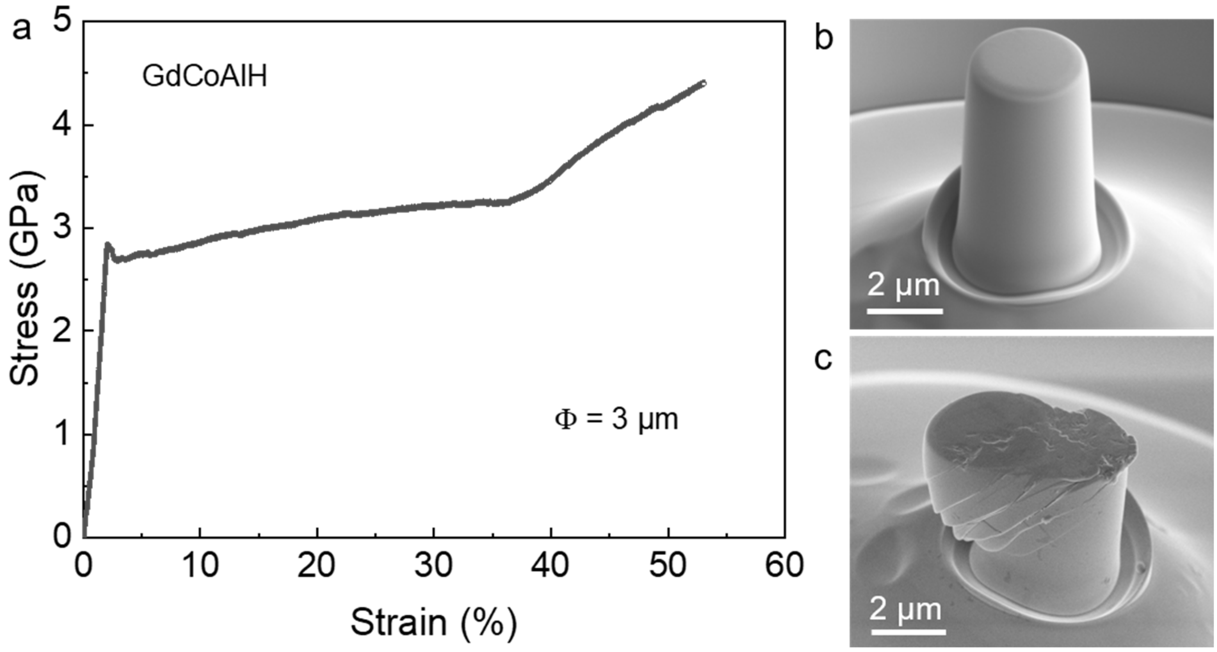

Supplementary Figure 3. Mechanical property of the GdCoAlH micropillars. **a** Engineering stress-strain curves of the GdCoAlH micropillar with a diameter (Φ) of 3 μm and height of 6 μm. **b**, **c** SEM images of the pillar before and after compression.

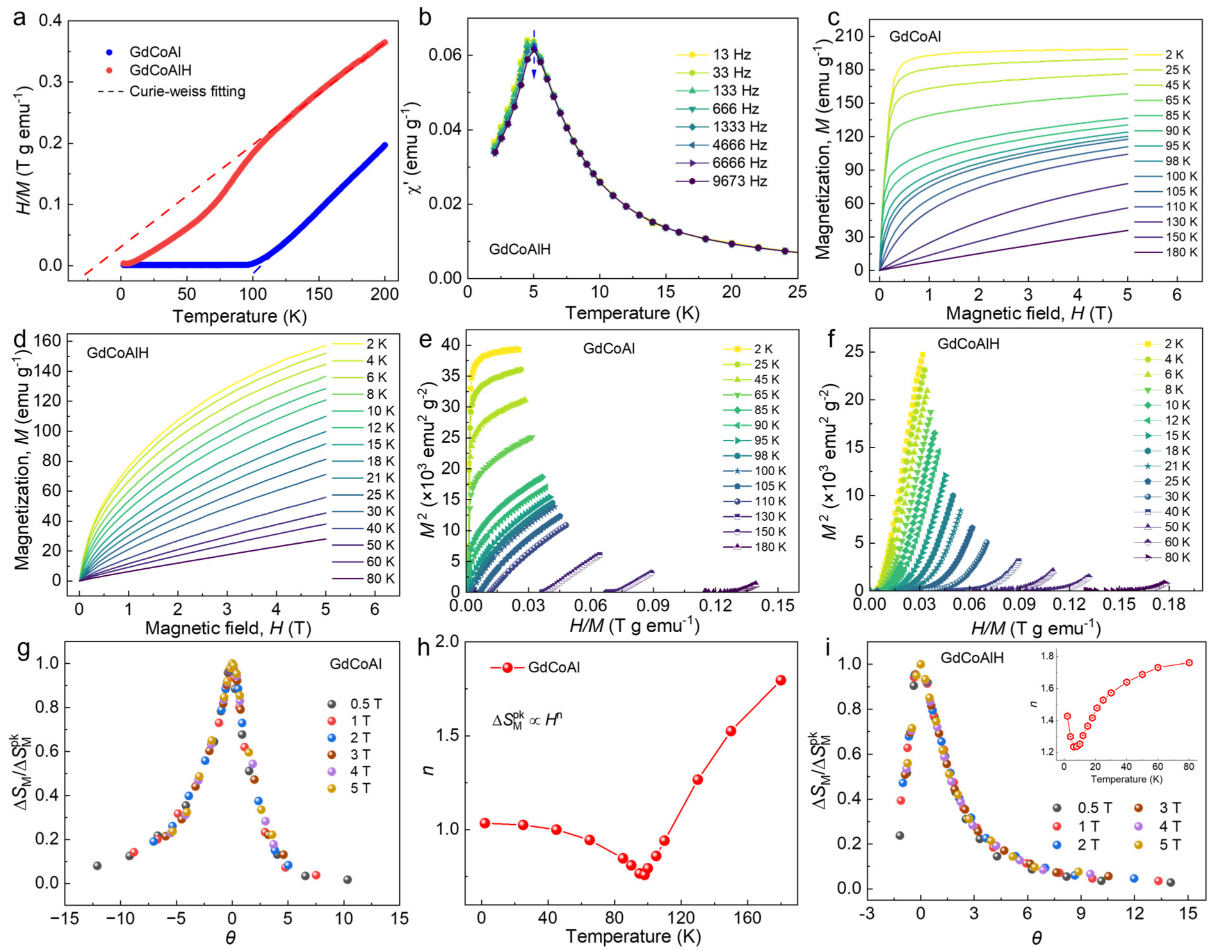

Supplementary Figure 4. Magnetocaloric measurements of the GdCoAl(H) powders. **a** Curie-Weiss fitting of the magnetization (*M*)-temperature curves. **b** Temperature dependence of AC magnetic susceptibility (real part, χ') varying from 13 to 9673 Hz for the GdCoAlH powder. **c**, **d** Isothermal magnetization curves and **e**, **f** corresponding Arrott curves. **g** The universal curves of magnetic entropy change (ΔS_M) and **h** temperature dependence of n value for the GdCoAl amorphous powder. **i** The universal curves of ΔS_M and n value of the GdCoAlH powder.

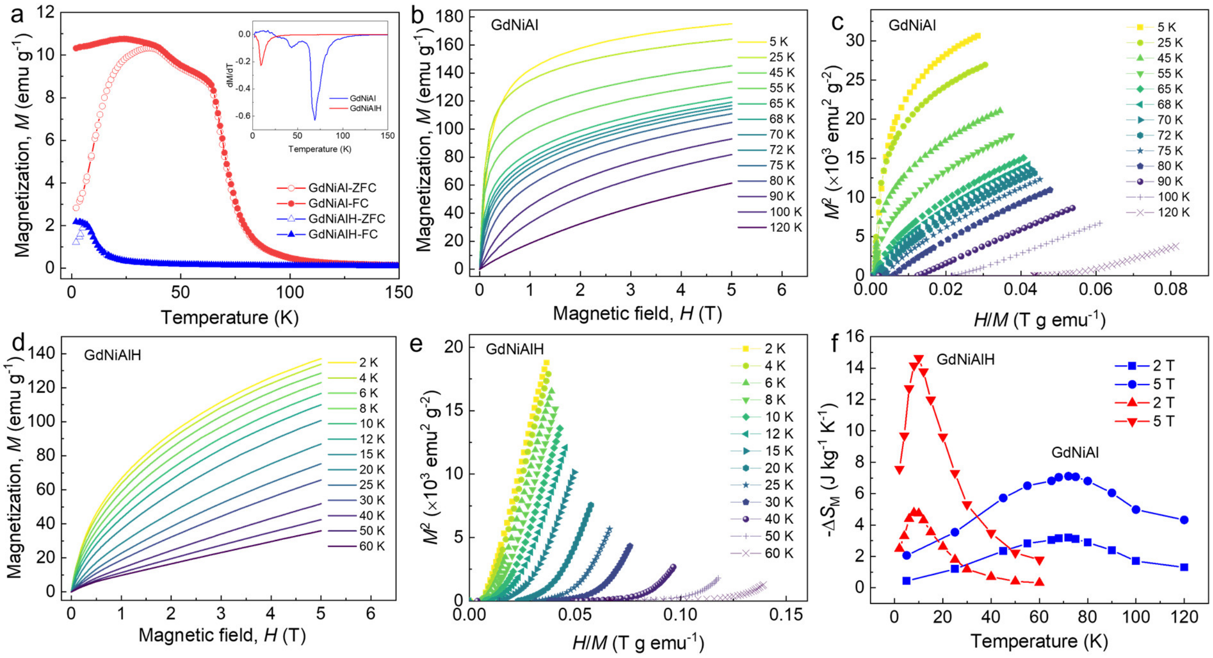

Supplementary Figure 5. Magnetocaloric measurements of the GdNiAl(H) powders. **a** Temperature dependence of magnetization under the applied magnetic field of 0.01 T. The inset shows the determination of Curie Temperature (T_C). **b**, **c** Isothermal magnetization curves and corresponding Arrott curves of the GdNiAl amorphous powders. **d**, **e** Isothermal magnetization curves and corresponding Arrott curves of the GdNiAlH powders. **f** Temperature dependence of ΔS_M under the maximum applied field of 2 and 5 T.

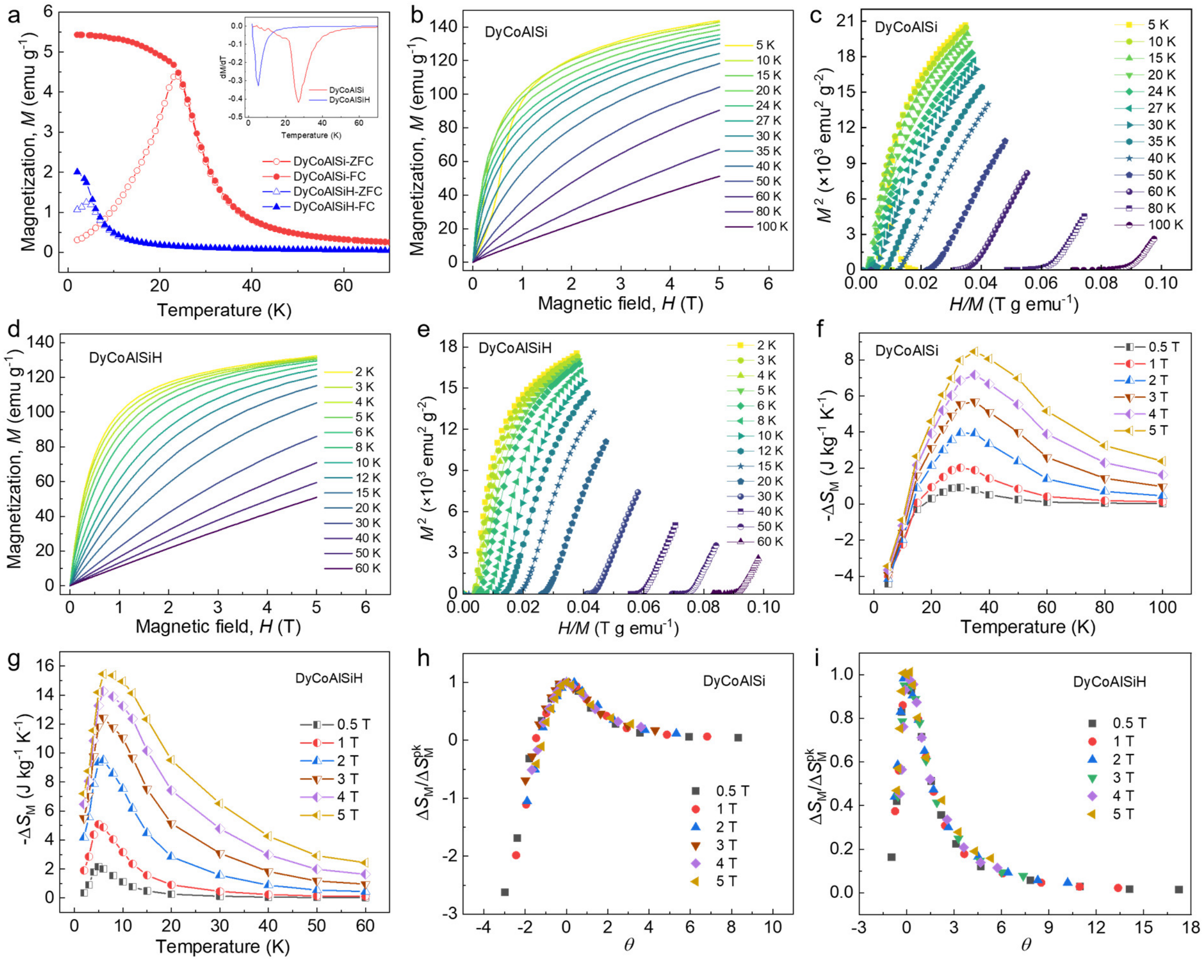

Supplementary Figure 6. Magnetocaloric measurements of the DyCoAlSi(H) powders. **a** Temperature dependence of magnetization under the applied magnetic field of 0.01 T. The inset shows the determination of T_C. **b**, **c** Isothermal magnetization curves and corresponding Arrott curves of the DyCoAlSi amorphous powders. **d**, **e** Isothermal magnetization curves and corresponding Arrott curves of the DyCoAlSiH amorphous powders. **f**, **g** Temperature dependence of ΔS_M under the maximum applied field from 0.5 to 5 T. **h**, **i** The universal curves of ΔS_M.

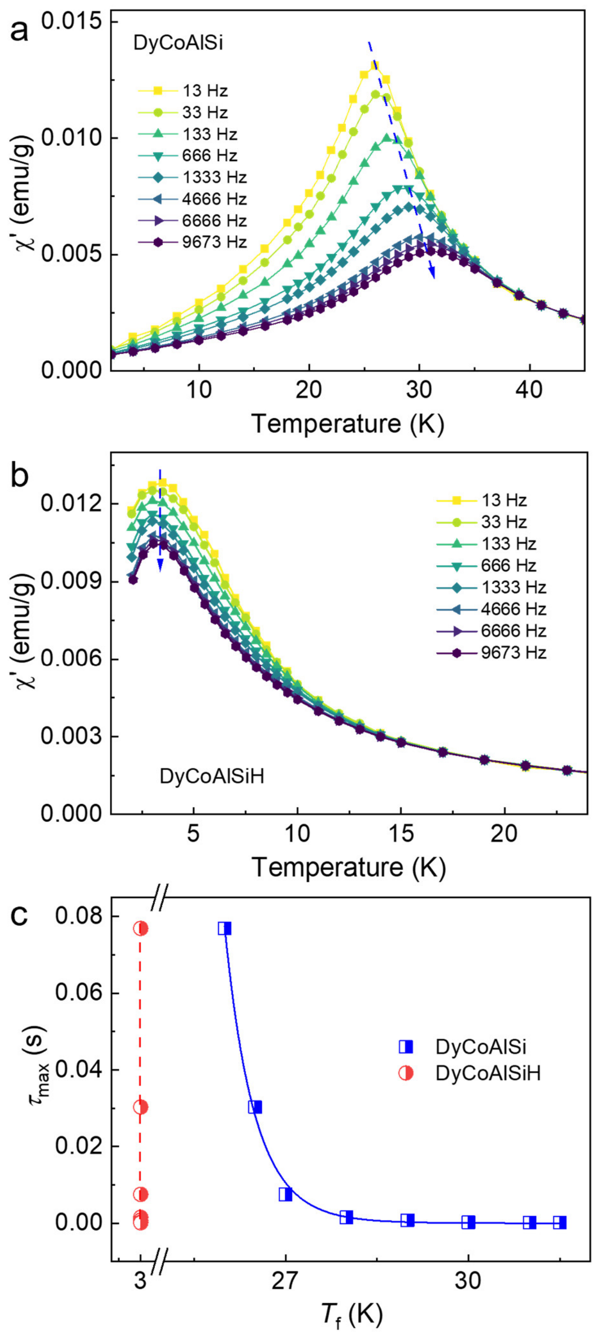

Supplementary Figure 7. Magnetic behavior of the DyCoAlSi(H) powders. The real part of magnetic susceptibility (χ') at frequency ranging from 13 to 9673 Hz for the **a** DyCoAlSi and **b** DyCoAlSiH powders. **c** The maximum relaxation time (τ_{max}) versus the peak temperature of magnetic susceptibility (T_f) for the DyCoAlSi and DyCoAlSiH powders.

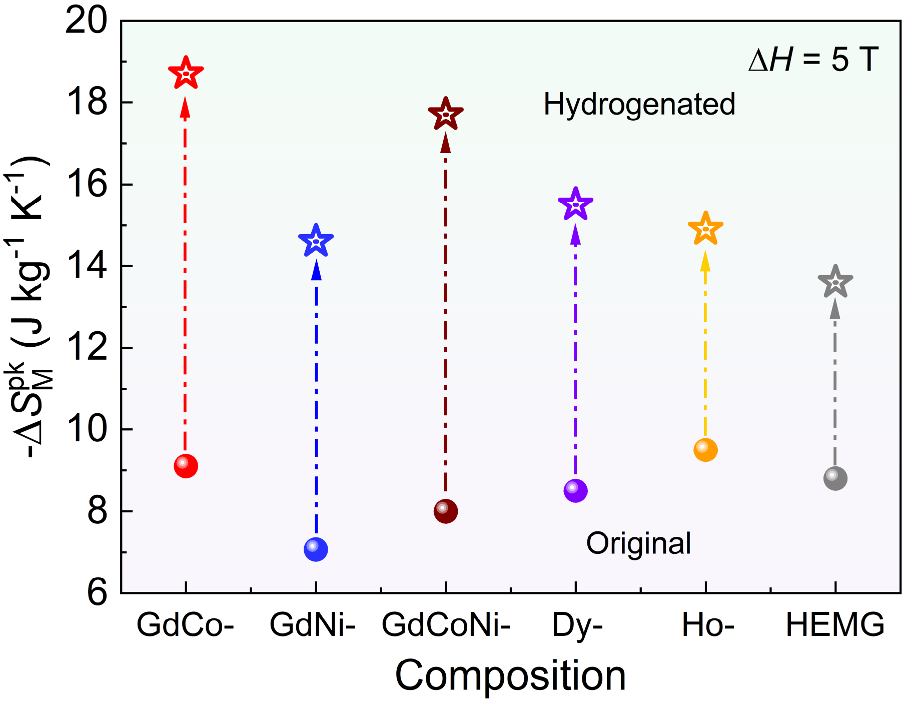

Supplementary Figure 8. Universality of hydrogenation to improve magnetic entropy change (ΔS_M). Comparation of maximum magnetic entropy change ($-\Delta S_M^{pk}$) (ΔH = 5 T) for the GdCo-, GdNi-, GdCoNi-, Dy-, Ho-based and high-entropy (HE, GdTbDyCoAl) MGs before and after hydrogenation.

.

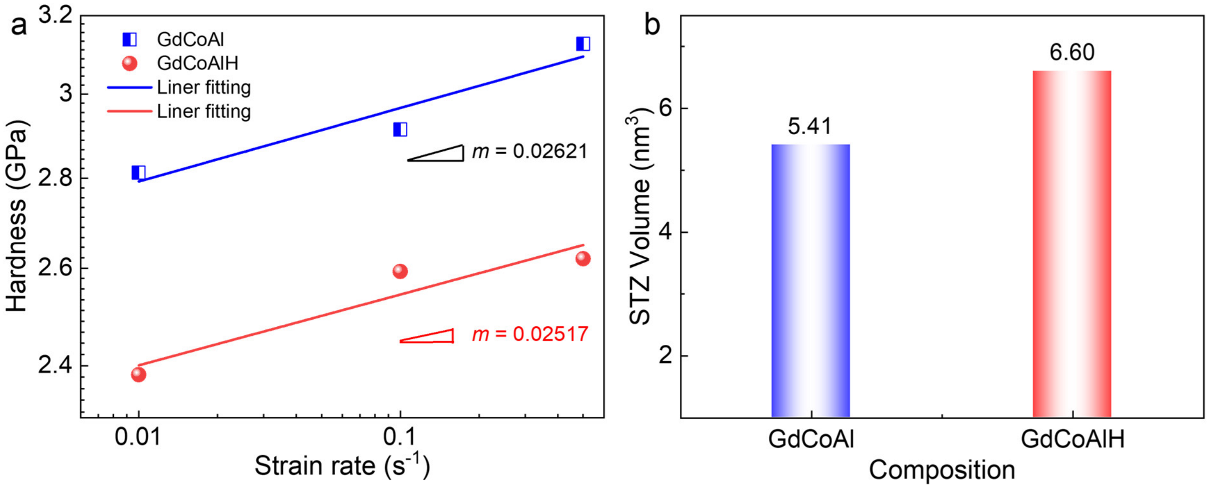

Supplementary Figure 9. Nanoindentation test of the GdCoAl(H) powders. **a** Strain rate dependence of hardness for the two samples **b** Shear transition zone (STZ) volume of the two samples.
